# Electric signals counterbalanced posterior vs anterior PTEN signaling in directed migration of *Dictyostelium*

**DOI:** 10.1186/s13578-021-00580-x

**Published:** 2021-06-14

**Authors:** Bing Song, Yu Gu, Wenkai Jiang, Ying Li, Wayne Nishio Ayre, Zhipeng Liu, Tao Yin, Christopher Janetopoulos, Miho Iijima, Peter Devreotes, Min Zhao

**Affiliations:** 1 School of Dentistry, College of Biomedical and Life Sciences, Cardiff University, Cardiff, CF14 4XY UK; 2 State Key Laboratory of Military Stomatology & National Clinical Research Center for Oral Diseases, School of Stomatology, Fourth Military Medical University, Xi’an, China; 3 Chinese Academy of Medical Sciences & Peking Union Medical College Institute of Biomedical Engineering, Tianjin, China; 4 BioImaging Core Facility, University of Sciences, Philadelphia, PA 19104 USA; 5 School of Medicine, Johns Hopkins University, Baltimore, MD 21205 USA; 6 Department of Dermatology, School of Medicine, University of California, Davis, CA 95618 USA

**Keywords:** PTEN, PIP3, Myosin, PH-Crac, F-actin, Electric field, Cell migration, Electrotaxis, Galvanotaxis

## Abstract

**Background:**

Cells show directed migration response to electric signals, namely electrotaxis or galvanotaxis. PI3K and PTEN jointly play counterbalancing roles in this event via a bilateral regulation of PIP3 signaling. PI3K has been proved essential in anterior signaling of electrotaxing cells, whilst the role of PTEN remains elusive.

**Methods:**

Dictyostelium cells with different genetic backgrounds were treated with direct current electric signals to investigate the genetic regulation of electrotaxis.

**Results:**

We demonstrated that electric signals promoted PTEN phosphatase activity and asymmetrical translocation to the posterior plasma membrane of the electrotaxing cells. Electric stimulation produced a similar but delayed rear redistribution of myosin II, immediately before electrotaxis started. Actin polymerization is required for the asymmetric membrane translocation of PTEN and myosin. PTEN signaling is also responsible for the asymmetric anterior redistribution of PIP3/F-actin, and a biased redistribution of pseudopod protrusion in the forwarding direction of electrotaxing cells.

**Conclusions:**

PTEN controls electrotaxis by coordinately regulating asymmetric redistribution of myosin to the posterior, and PIP3/F-actin to the anterior region of the directed migration cells.

**Supplementary Information:**

The online version contains supplementary material available at 10.1186/s13578-021-00580-x.

## Introduction

Physiological electric signals function as one of the vital guidance cues for directed cell migration during wound healing, development and regeneration of multicellular organisms. To date, many cell types have shown directed cell migration response to applied electric fields (EFs), namely electrotaxis or galvanotaxis [1–9]. Several hypotheses have been tested in the past to compare the molecular basis of electrotaxis with chemotaxis, including excitation of membrane potential leading to biased activation of voltage-gated ion channels, electrostatic and electro-osmotic forces at the plasma membrane triggered redistribution of charged membrane components, etc. [2, 6, 10]. EF stimulation affects the membrane potential of a variety of cells [11–14]. Our previous studies that reduced membrane potential diminished electrotaxis without affecting chemotaxis [2], and G-protein coupled receptors pivotal for chemotaxis was marginally required in electrotaxis [7], indicate that the mechanisms regulating electrotaxis defer at least in part from chemotaxis. The facts that electrotaxis is sensitive to pH change [10], and depolarized membrane potential only partially reduced the electrotactic response [2], suggesting that alternative mechanisms including the redistribution of membrane components could play a more crucial role in electrotaxis. Indeed, our previous studies proved that EF could drive a variety of growth factor receptors and membrane lipids to redistribute asymmetrically, leading to polarized PI3K vs PTEN signaling in electrotaxing cells [1, 4, 8, 9]. Asymmetric PI3K redistribution to the leading edge of the cells has been shown to play an important role in the control of electrotaxis [9]; however, the mechanistic regulation of PTEN in electrotaxis remains elusive.

Extensive evidence indicated that PTEN is critical in the regulation of cell migration [15–20], and enriched asymmetrically at the back and sides of the directionally migrating cells in chemoattractant gradients [16, 21]. Being a multifunctional enzyme as dual protein and lipid phosphatase, PTEN dephosphorylates PtdIns(3,4,5)P_3_ to inactivate downstream signaling in wild type cells [22, 23]. *Pten* knockout triggered a significant increase of both PI(3,4,5)P3 and protein kinase B (Akt) activation, which can be rescued by re-expression of *pten* [24–33].

Although the mechanism is not entirely clear, increasing studies suggested that PTEN regulates directed cell migration in association with PI3K signaling. *Pten* knockout or knock-in *Dictyostelium* cells exhibit decreased motility and chemotaxis defect due to the altered basal activity of the PI3K pathway [21, 34]. PI3K and PTEN coordination is required for shaping the temporal and spatial localization of PIP_3_ during chemotaxis [35]. In chemotaxing neutrophils and *Dictyostelium* cells, PI3K recruits to the leading edge, and PTEN localizes at the sides and rear to reinforce anterior PIP3 accumulation [36, 37]. PTEN Posterior localization was associated with the anterior PIP3 activity through either phosphatase activity-dependent mechanism where PIP3 induced membrane dissociation of PTEN at the leading edge of chemotaxing cells, or phosphatase activity-independent mechanism where PIP3 induced reduction of the membrane binding sites for PTEN [38]. PH domain of Cytosolic regulator of adenylyl cyclase (PH-Crac) binds to PIP3 and asymmetrically redistributes to the leading edge of the migrating cells, which can be used as an indicator for PIP3 signaling [39].

Actin and myosin II are major structural and force-generating components of chemotaxis machinery [40, 41]. PTEN is an upstream component essential for the relocalization of myosin II and F-actin to the cortex, which is required for the suppression of lateral pseudopod formation during chemotaxis [42, 43]. PTEN and myosin II were reported to colocalize and redistribute toward the posterior region of directionally migrating cells, which confers posterior contraction [18, 21, 36, 41, 44–47]. Myosin II null cells could not suppress rear and lateral pseudopod formation or form polarity and showed chemotaxis inefficiency [48, 49]. PTEN is responsible for maintaining persistent F-actin activity since *pten* null cells showed an increased number of short-lived F-actin protrusions in chemotaxis [50]. PTEN is also countable for the coordination of anterior signaling of PI3K/Akt and actin polymerization in chemotaxing cells [34].

Our previous studies demonstrated that EFs triggered asymmetric redistribution of PI3K/Akt to the leading edge of directionally migrating cells independent of actin polymerization [9], and both PIP3 and F-actin colocalized at the anterior region of the electrotaxing cells [4]. In this study, we aim to elucidate the role of PTEN signaling in electrotaxis through the regulation of myosin II vs F-actin translocation towards the posterior vs anterior plasma membrane, respectively. Here we show that in *Dictyostelium* cells, asymmetric posterior membrane translocation of PTEN is required in the regulation of electrotaxis. Such asymmetric PTEN redistribution is an earlier event responsible for the posterior translocation of myosin II and anterior relocalization of PIP3/F-actin, thereby generates a biased pseudopod production in the forwarding direction to facilitate the electrotaxis of *Dictyostelium* cells.

## Materials and methods

### Cell culture and development

Wild-type (AX2) and *pten*^−^ cells were cultured axenically on tissue culture Petri dishes in HL5 medium and shaking suspension containing HL5-glucose medium (200 rpm) at 22 °C. PTEN-GFP/WT, PTEN-GFP/*pten*^−^, myosin II- GFP/WT and myosin II-GFP/*pten*^−^, were maintained in culture medium containing 20 μg/ml G418. All strains were starved for 1 h in development buffer, then pulsed with 100 nM cAMP every 6 min in shaken suspension (150 rpm) for 5 h before experiments.

### Electrotaxis assay

Developed cells were seeded in a specially constructed trough either on cell culture dishes or glass slides. Direct current electric fields of indicated strengths were applied in custom-designed electrotaxis chambers as described previously [7, 51]. In control experiments, cells were pretreated in 2 mM caffeine to exclude the adenylate cyclase activation triggered cell-to-cell cAMP signaling, and tested in the custom-made continuous perfusion electrotaxis chamber to rule out the potential chemical gradients build up generated by EF stimulation.

Bright field time-lapse images were captured at room temperature using DeltaVision™ imaging system with a motorized X, Y, Z stage (IMSOL, UK), at a time interval of 20 s per frame. The directedness of cell migration was assessed using the formula Σcosθi/n, where θi was the angle between the EF vector and the direction of the cell movement for an individual cell from a cluster of cells, and n was the total number of cells. We named the “anode to cathode” direction the positive migration direction.

A cell moving directionally toward the cathode would have a θ angle of 0 and a directedness of 1 (cos ((0π)/180) = 1); a cell moving directionally toward the anode would have a θ angle of 180 and a directedness of − 1 (cos ((180 π)/180) = − 1). An overall directedness value approaching 1 (or − 1) indicates directional migration in the EF to the cathode (or anode), whereas an overall directedness close to zero indicates random migration. Trajectory speed is the total distance travelled by a cell divided by time. Displacement speed is the straight-line distance from starting and ending points of a cell divided by the time.

### PTEN, myosin, and PH-Crac distribution analysis with fluorescence microscopy

Cells expressing PTEN-GFP, myosin II-GFP or PHCrac-GFP constructs were pretreated with 20 µM MG132 in development buffer (DB) to block proteasomal degradation and placed on a custom-designed electrotaxis chamber as described previously [51]. To visualize GFP fused proteins, we observed the cells using an Ultraview confocal microscope (PerkinElmer, USA) and DeltaVision™ imaging system (IMSOL, UK) coupled with an inverted microscope and a CoolSNAP EZ camera, at a time interval of 20 s per frame. The distribution of PTEN-GFP, myosin II-GFP and PHCrac-GFP was measured using the linear fluorescence scan function of ImageJ software. To quantify the fluorescence recruitment of PTEN-GFP, myosin II-GFP and PHCrac-GFP to the plasma membrane of the cells, we measured fluorescence intensities in 1-pixel areas and averaged the results of 5 randomly chosen positions in both posterior and anterior regions of the plasma membrane. Similar measurements were conducted from 10 randomly picked positions in the cytosol to generate the relative fluorescence intensity (plasma membrane/cytoplasm) in the presence or absence of EF treatment. Background fluorescence intensity was subtracted from all measurements.

To further demonstrate the EF-triggered asymmetric redistribution of PTEN-GFP, myosin II-GFP and PHCrac-GFP to the plasma membrane of the electrotaxing cells, the fluorescence intensity ratio analysis was conducted using the Integrated Density function of ImageJ software. Identical areas of 2 × 2 pixels were randomly chosen at the anterior and posterior plasma membrane of the cells, from at least five different plasma membrane sites in each cell using at least 50 cells from minimal three independent experiments. The integrated mean density of the fluorescence intensities within the areas was measured, and background signals were subtracted before conducting the posterior vs anterior (for PTEN-GFP and myosin II-GFP) or anterior vs posterior (PH-Crac) ratio analysis.

### Immunocytochemistry staining

For immunocytochemistry staining, cells were fixed using 1% PFA and stained for PTEN-GFP (rabbit anti-GFP, Millipore, AB3080), phospho-PTEN (Ser380/Thr382/383, Cell Signaling, Cat#9549), myosin II (mouse mAb 56–396-5), PHCrac-GFP (Mouse mAb anti-GFP, Thermo Fisher, A-11120 and F-actin (TRITC-phalloidin). Fixed cells were imaged using the DeltaVision™ imaging system (IMSOL, UK) with a 63 × objective. Images were processed with ImageJ.

### Immunoblot analysis of PTEN, myosin II and PH-Crac membrane translocation

Cells were pretreated with caffeine, then washed, and resuspended in PM buffer (Phosphate magnesium buffer, pH 6.5. 5 mM Na2HPO4, 5 mM KH2PO4, 2 mM MgSO4), before seeded in a custom-designed electrotaxis chamber. At indicated time points after EF stimulation, either whole cells or membrane fractions of cells were filter-lysed into ice-cold PM buffer to terminate any potential ripple effect post EF treatment. Membrane fractions were collected by centrifugation at 15,000×*g* for 1 min and assayed by immunoblot of anti-GFP antibody (mouse mAb anti-GFP, Thermo Fisher, A-11120). Phospho-PTEN expression was also examined in the same way above, using Phospho-PTEN antibody (Ser380/Thr382/383; Rabbit mAb, Cell Signaling, Cat#9549).

### Phosphatase activity analysis of PTEN

The PTEN phosphatase activity was determined quantified as described previously (Nguyen et al. 2014). PTEN-GFP was immunopurified using GFP-Trap agarose beads (Allele Biotech) on *Dictyostelium* cells. The phosphatase activity was quantified by measuring the release of phosphates from PI(3,4,5)P3 diC8 (Phosphatidylinositol 3,4,5-trisphosphate diC8), using a Malachite Green Phosphatase assay kit (Echelon Biosciences).

### Pseudopod protrusion analysis

Pseudopod protrusions were analyzed with ImageJ. A new pseudopod was defined as either daughter protrusions splitting from their parent in the previous frame or a new lateral pseudopod that did not have a parent. The centroid and center point of the plasma membrane base of pseudopod protrusion were calculated with ImageJ. The orientation of new pseudopods was calculated as the direction of the line between the pseudopod centroid and the center point of plasma membrane base of the pseudopod with respect to the EF vector. Only active pseudopods were included in the study.

### Statistic analysis

Data are reported as mean ± SEM, with n denoting the number of tests or the number of cells for the migration assay. Means were compared using one-way analysis of variance (ANOVA) in group comparison. A two-tailed Student’s t-test for unpaired data was applied as appropriate. A value of P < 0.05 was considered statistically significant.

## Results

### PTEN is required in the electrotactic response of *Dictyostelium*

**Fig. 1:**
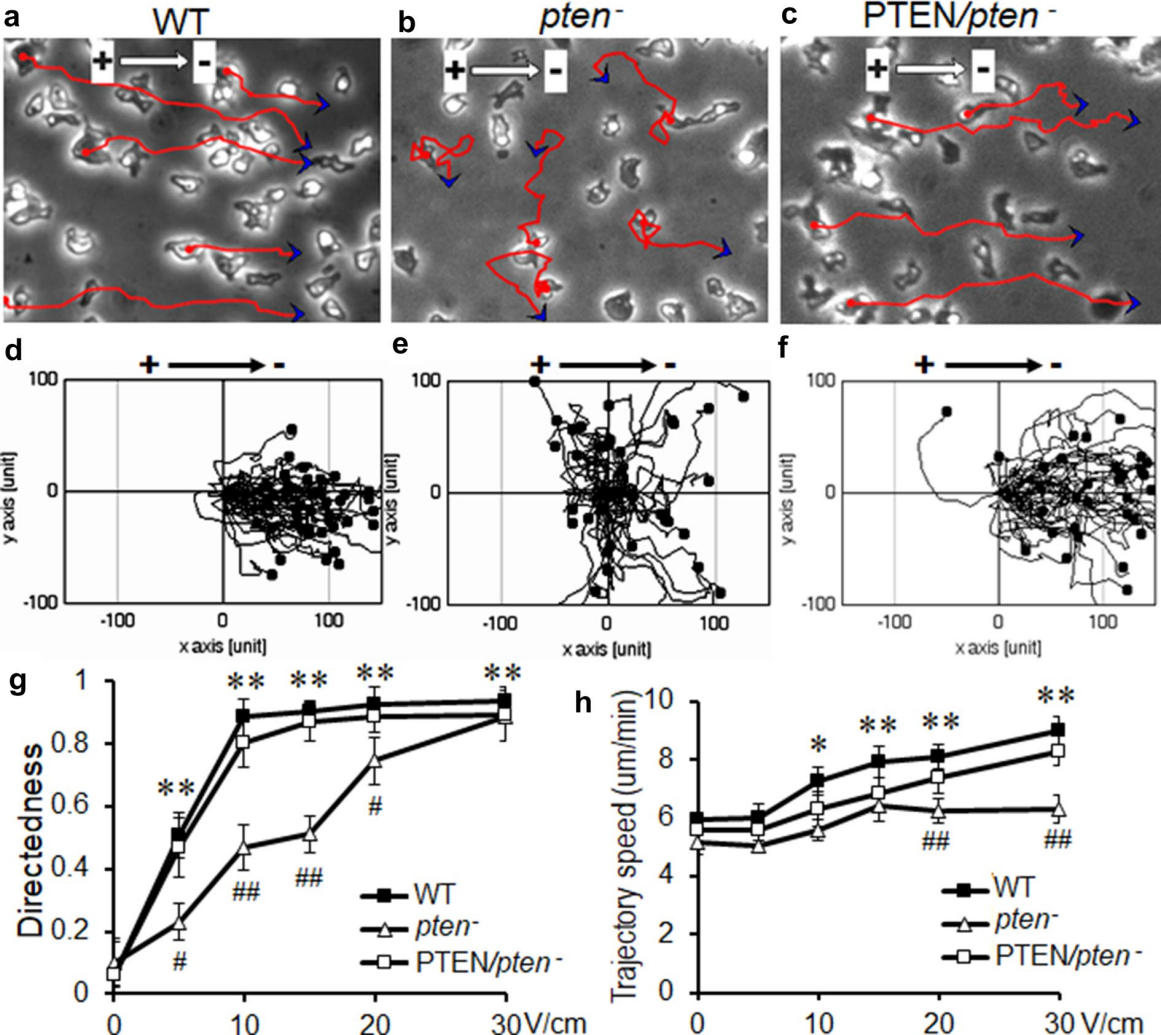
*pten* null mutation significantly reduced electrotaxis at lower voltages, which was recovered by re-expression of *pten*. **a-c** Images selected from time-lapseVideos showing the migrating cells. Red lines and blue arrows are cell trajectories and endpoints over 15 min. **d-f** Composite trajectories of migrating cells with the starting points placed at the origin. **b** and **e** *pten*^−^ cells showed significantly reduced electrotaxis. **c** and **f** The re-expression of wild type *pten* on *pten*^*−*^ (PTEN/*pten*^*−*^, or *pten* rescue) completely reversed the electrotaxis defects in the *pten*^*−*^ cells. **g**, **h** voltage dependence migration directedness and trajectory speed. *, P < 0.05; **, P < 0.01, compared with no EF control; #, P < 0.05; ##, P < 0.01 compared between WT/*pten* rescue groups and *pten*^*−*^ group, one-way ANOVA. A minimal 150 cells were analyzed from each experimental group. A minimal 3 repeats were conducted for all conditions investigated. EF = 10 V/cm in **a–f**, with cathode to the right

In the absence of EF, AX2 wild type (WT) cells moved randomly with the migration directedness close to zero (Fig. 1g). EFs triggered an obvious electrotactic response of *Dictyostelium* cells toward the cathode in a voltage-dependent manner (Fig. 1a, d and g; Additional file 1: Video S1). 5 V/cm EF treatment triggered a clear electrotactic response of WT cells toward cathode when compared with non-treated cells (Fig. 1g, p < 0.05, one-way ANOVA). The electrotactic response of WT cells maximized at 10 V/cm EF, with migration directedness approaching 1, which indicates that the majority of the cells migrate directionally towards the EF vector (Fig. 1g).

Compared with WT cells, *pten*^*−*^ cells showed significantly reduced electrotactic response when treated with EFs between 5 and 15 V/cm (Fig. 1b, e and g, Additional file 2: Video S2; P < 0.05 for 5 V/cm, P < 0.01 for 10 and 15 V/cm, one-way ANOVA). The electrotactic response of *pten*^*−*^ cells gradually elevated at 20 V/cm and was fully restored to WT cells level when treated with 30 V/cm of EF (Fig. 1g). PTEN re-expression in *pten*^*−*^ cells (PTEN/*pten*^*−*^, or *pten rescue*), on the other hand, completely reinstated the reduced electrotactic response of *pten*^*−*^ cells back to WT cells level under all experimental conditions (Fig. 1c, f and g; Additional file 3: Video S3). EF treatment at 10 V/cm or higher significantly increased the trajectory speed of both WT and PTEN/*pten*^*−*^ cells, which dictates increased motility of the migrating cells in EFs (Fig. 1h). In contrast, disrupting PTEN abolished the EF-promoted motility enhancement at 20 and 30 V/cm (Fig. 1h; ##, P < 0.01, one-way ANOVA). Putting together, the data above suggest that PTEN is required for electrotaxis of *Dictyostelium*.

### Electrotaxis of *Dictyostelium* cells requires asymmetrical plasma membrane translocation and activation of PTEN

**Fig. 2:**
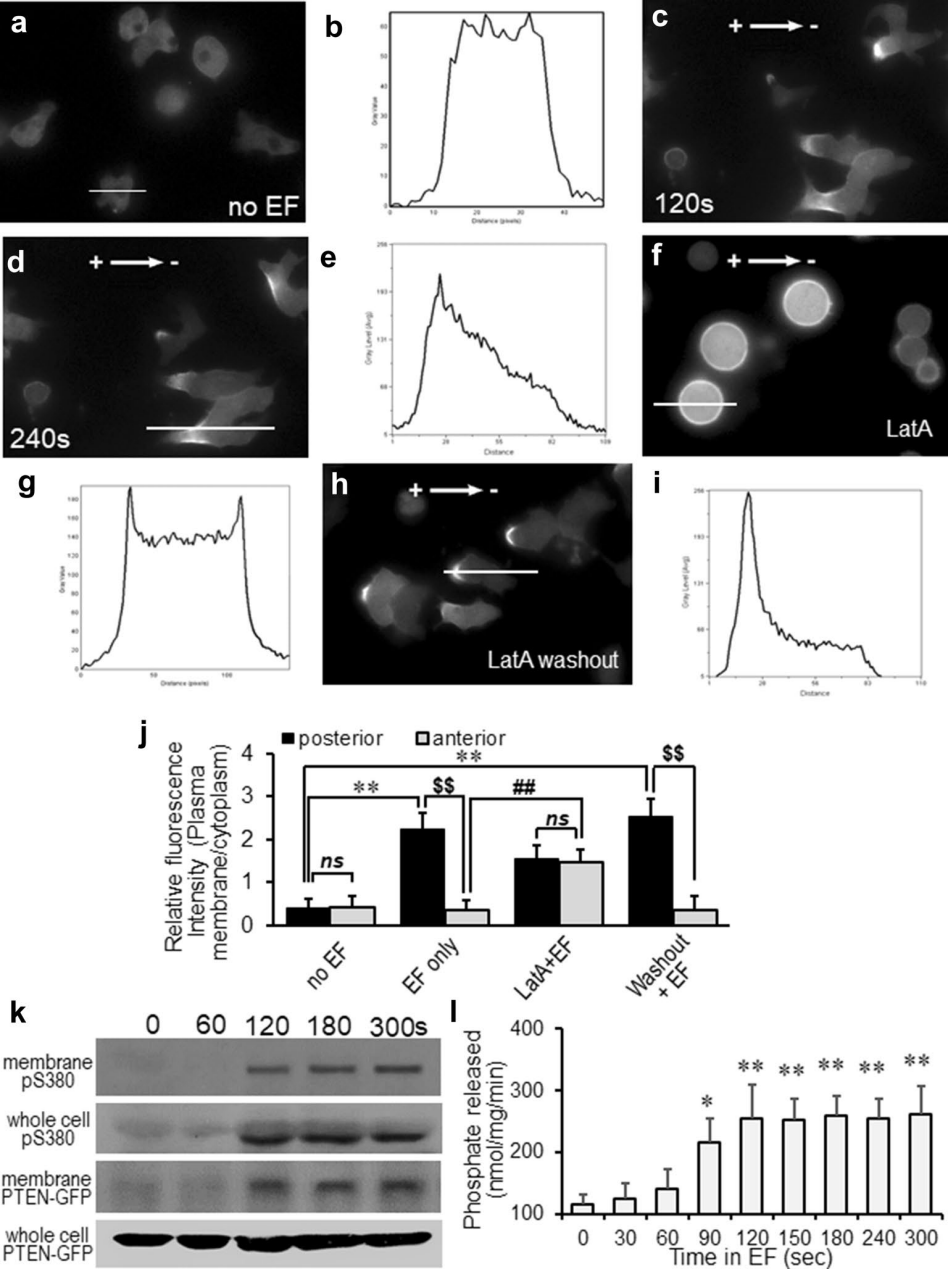
PTEN-GFP redistributed to the posterior plasma membrane of electrotaxing cells, which is depended on actin polymerization. **a**, **b** *Dictyostelium* cells did not show the membrane recruitment of PTEN-GFP in the absence of EF. (**c–e**) An applied EF induced asymmetrical redistribution of PTEN-GFP to the posterior of *Dictyostelium* cells. (**f** and **g**) Latrunculin A treatment abolished asymmetrical redistribution of PTEN, while plasma membrane recruitment was still maintained (see J, ##: P < 0.01, compared between Latraculin A treated and control. **h** and **i** Washout latrunculin A restored the asymmetrical redistribution of PTEN. **b, e, g and i** representative line scan of fluorescence intensity of PTEN-GFP for marked cells in **a, d, f and h**, respectively. (**j**) GFP intensities at the plasma membrane were determined relative to that in the cytosol. Values represent the mean ± s.d. **, P < 0.01 compared between the posterior membrane of no EF vs EF treated group; $$, P < 0.01 compared between posterior and anterior membrane within EF treated cells; ##, P < 0.01 compared between anterior within “LatA + EF” and “EF only” group; *ns*, P > 0.05 compared between the posterior and anterior membrane of “no EF” and “LatA + EF” group. **k** Membrane fraction and the whole-cell lysates from cells treated with the indicated duration of EFs were analyzed by immunoblotting with antibodies against phospho-PTEN (pS380) or GFP (PTEN-GFP), respectively. **l** The PTEN proteins were immunopurified from *Dictyostelium* cells treated with varies duration of EF, and phosphatase activities were measured. *P < 0.05; **P < 0.01 compared with no EF group. n ⩾ 3

Using human PTEN-GFP expressing *Dictyostelium* cells, we monitored the dynamic plasma membrane translocation and redistribution of PTEN during electrotaxis. Similarly, as demonstrated in previous studies, asymmetric PTEN plasma membrane recruitment was not observed on *Dictyostelium* cells in the absence of EF [16, 52]. Compared with no EF control, which showed minimal plasma membrane translocation (Fig. 2a, b), EF stimulation triggered significantly more posterior plasma membrane translocation of PTEN-GFP than the anterior region (Fig. 2c, d, j, P < 0.01; Additional file 4: Video S4). GFP intensity line scan analysis confirmed the asymmetrical redistribution of PTEN-GFP at the posterior plasma membrane of the cells (Fig. 2e). Fluorescence intensity ratio analysis (posterior vs anterior plasma membrane) further revealed that the PTEN plasma membrane translocation and posterior redistribution started at 42 ± 9.4 s and peaked at 119 ± 10.7 s post EF stimulation (Fig. 4a, red line). Latrunculin A (LatA) treatment completely abolished the EF-triggered PTEN posterior plasma membrane redistribution and electrotaxis of the cells (Fig. 2f, g; Additional file 5: Video S5), which is a fully reversible event when LatA was washed out (Fig. 2c, d, g, h and i; Additional file 6: Video S6). Interestingly, although LatA fully abolished EF-triggered posterior redistribution of PTEN-GFP (Fig. 2h, P > 0.05 between posterior and anterior within “LatA + EF” group), it did not affect the plasma membrane translocation of PTEN in EF (Fig. 2j, ## P < 0.01 compared between “LatA + EF” and anterior of “EF only” group). The fact that blocking actin function triggered significantly elevated but even translocation of PTEN-GFP to the plasma membrane, suggesting that PTEN mediated electrotaxis requires actin polymerization via maintaining the biased PTEN signals to the rear region of directionally migrating cells. PTEN mediated electrotaxis was further supported by the following observations: 1). membrane fraction of PTEN expression was detectable as early as 120-s post EF stimulation (Fig. 2k). 2). EF-triggered PIP3-C8 phosphatase activity peaked at 120-s post EF treatment (Fig. 2l). 3). Activation of PTEN phosphorylation from both plasma membrane fraction and whole-cell lysate (Fig. 2k) also coincided with the PTEN posterior plasma membrane translocation at 120 s after EF exposure (Fig. 4a, red line). And 4). EF-triggered PTEN posterior relocalization preceded the electrotactic response of WT cells (Fig. 4b, red line, peaked at 200 s post-EF stimulation), which was abolished by PTEN knockout (Fig. 4b, blue line).

### PTEN-dependent posterior plasma membrane translocation of myosin II in EF

**Fig. 3:**
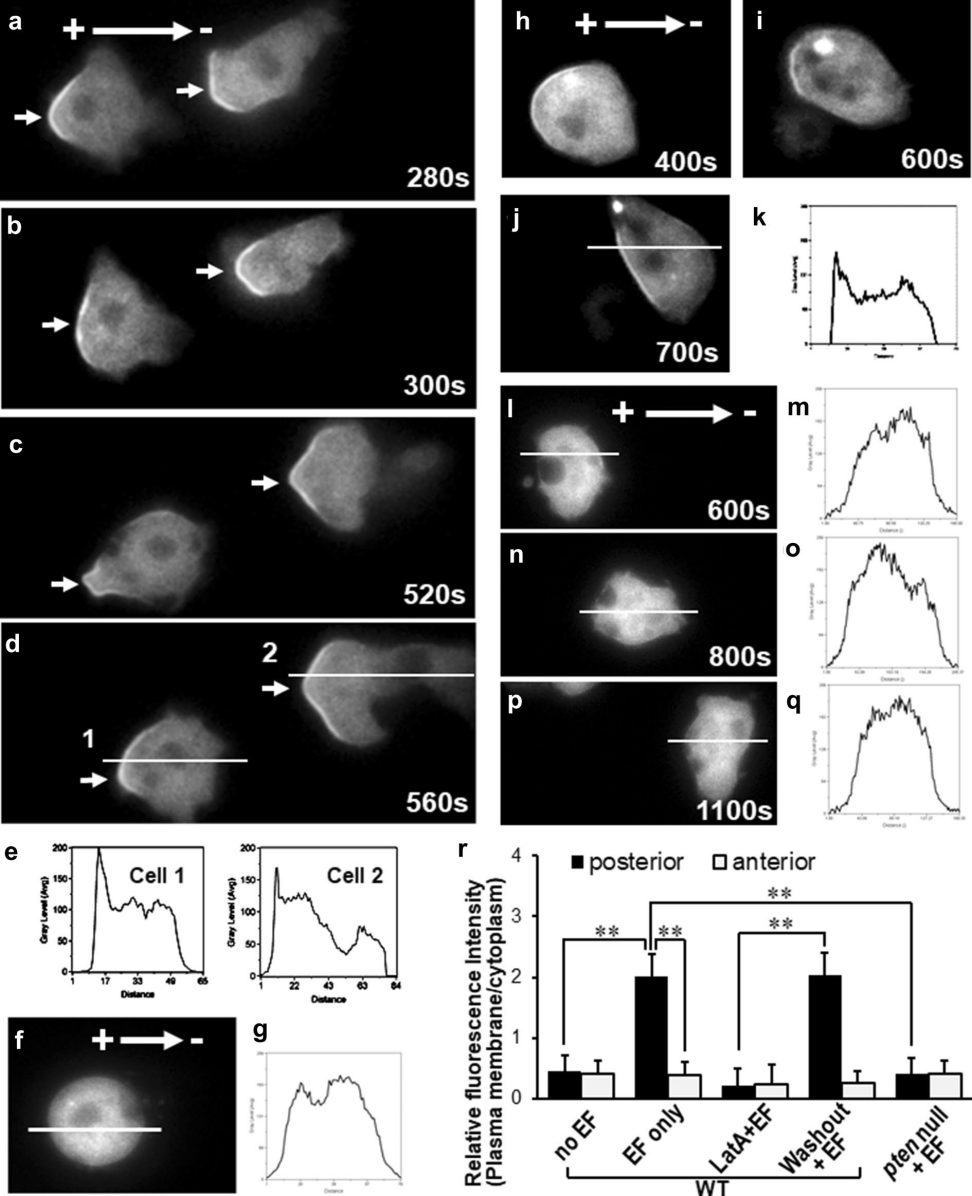
Myosin II redistributed to the posterior plasma membrane of electrotaxing cells, which is depended on actin polymerization and PTEN signaling. **a–e** Time-lapse images showing translocation and asymmetric redistribution of Myosin II to the posterior plasma membrane of the WT cells in EF (10 V/cm). **f, g** Latrunculin A treatment abolished asymmetric membrane translocation of myosin-GFP in EF. EF = 30 V/cm. Similar results were observed in 34 cells from a minimum of three independent experiments. **h–k** Washout of latrunculin A restored the asymmetrical redistribution of myosin GFP to the posterior plasma membrane of the electrotaxing WT cells. See Movie “Smovie11_myosinGFP_EF_LatA_washout”. EF = 30 V/cm. The same results were observed in 53 cells from 3 independent experiments. **l-q** Myosin-GFP/*pten*^*−*^ lost plasma membrane redistribution of myosin II in EF (30 V/cm). **e, g, k, m, o, q** fluorescence intensity line-scan of myosin-GFP along the lines indicated in **d, f, j, l, n and p**, respectively. **r** GFP intensities at the plasma membrane were determined relative to that in the cytosol. Values represent the mean ± s.d. (*n*⩾15)

**Fig. 4:**
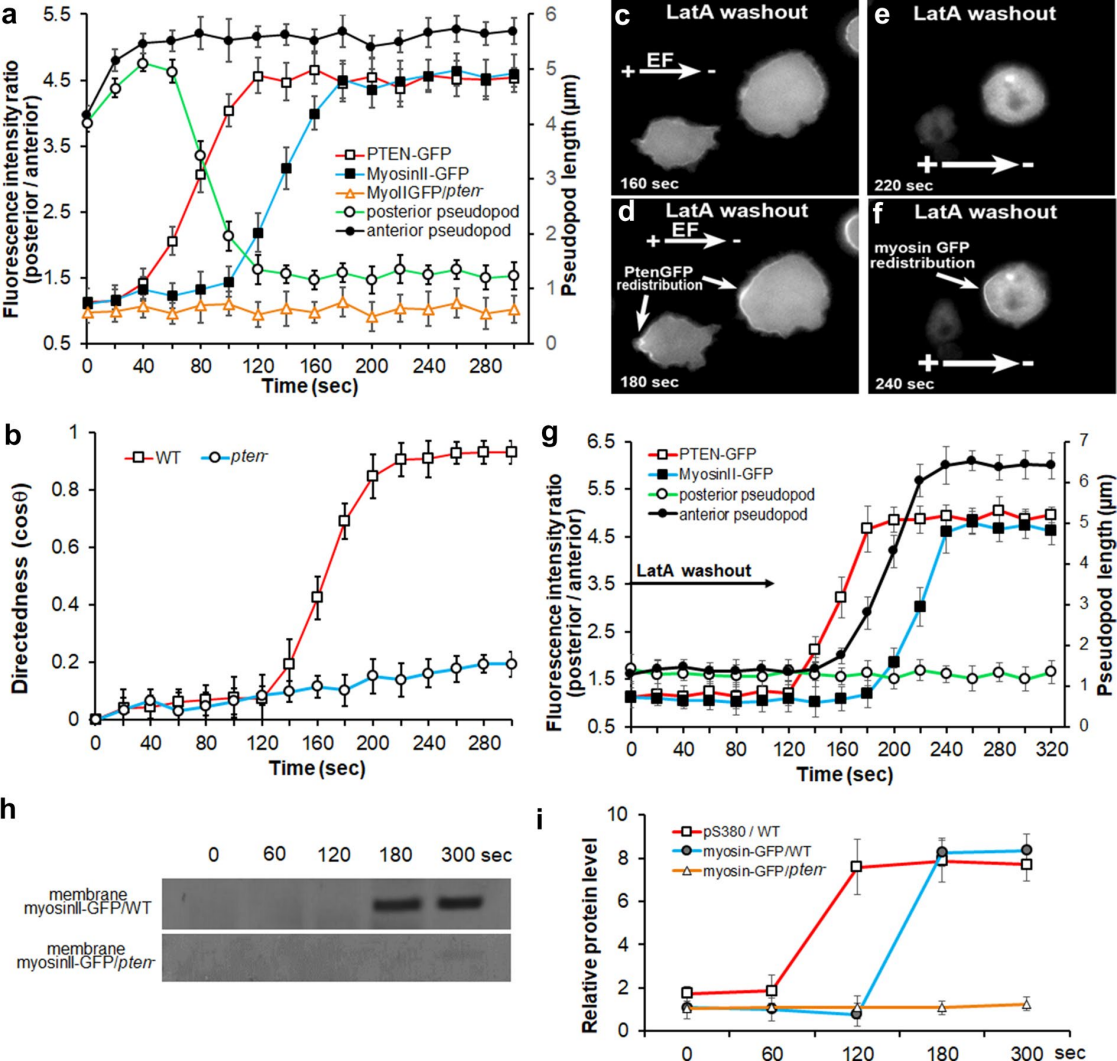
PTEN posterior membrane translocation proceeded that of myosin and the electrotactic response of the cells. EFs were applied at time zero in all experiments. **a** Time-lapse analysis of fluorescence intensity ratio comparing GFP translocation toward posterior vs anterior plasma membrane in EF. The asymmetric redistribution of myosin II-GFP (blue line) showed significant delay compared to that of PTEN-GFP (red line). Such asymmetric relocalization was fully abolished in *pten*^*−*^ cells (orange line). EF triggered a sharp shortening of the average pseudopod length at the posterior region (green line), while increased that at the anterior of the electrotaxing WT cells (black line). **b** Time-lapse analysis of the directional migration of WT (red line) and *pten*^*−*^ cells (blue line). EF = 10 V/cm. **c**-**f** Comparison of the re-establishment of the PTEN-GFP and myosin II-GFP posterior membrane translocation post latrunculin A washout in EF. **g** Time-lapse analysis of fluorescence intensity ratio comparing GFP translocation toward posterior vs anterior plasma membrane post latrunculin A washout in EF. The asymmetric redistribution of PTEN-GFP (red line) preceded that of myosin II-GFP (blue line). EF triggered elongation of the average pseudopod length was re-established at the anterior region (black line), but not at the posterior region of the cells (green line). **h** Membrane fraction cell lysates from the cells treated with the indicated duration of EFs were analyzed by immunoblotting with antibodies against plasma membrane fraction of myosin II. **i** Band intensity was quantified (n ≥ 3)

Myosin II is another posterior signaling regulator in close association with PTEN, which redistributed to the posterior plasma membrane of chemotaxing cells [42, 53]. To explore the role of myosin II in electrotaxis, we tested the dynamic redistribution of myosin and its correlation with PTEN during electrotaxis of *Dictyostelium* on myosin II-GFP/WT and myosin II-GFP/*pten*^*−*^ cells. Similar to the findings from PTEN-GFP, localized plasma membrane recruitment of myosin II was also detected at the rear of electrotaxing *Dictyostelium* when treated with EFs at 10 V/cm (Fig. 3a-e; 3R, P < 0.01 compared between posterior and anterior myosin II-GFP within the “EF only” group; Additional file 7: Video S7). Fluorescence intensity ratio analysis (posterior vs anterior plasma membrane) further revealed that the myosin II plasma membrane translocation and posterior redistribution started at 102 ± 8.9 s and peaked at 183 ± 11.3-s post EF stimulation (Fig. 4a, blue line). LatA-treatment abolished EF-triggered asymmetric redistribution of myosin II-GFP (Fig. 3f, g; Additional file 8: Video S8), suggesting myosin II relocalization during electrotaxis is actin-dependent. This is a reversible event since washout of latA fully restored the asymmetrical redistribution of myosin II-GFP to the posterior plasma membrane of the electrotaxing cells (Fig. 3h, k; 3R, P < 0.01 compared between “washout” and “LatA” groups; Additional file 9: Video S9). In contrast, *Pten* null cells lost posterior membrane translocation of myosin II-GFP in EF completely compared with WT cells evenly in the highest EF tested (Fig. 3l-q; 4A, orange line; 3R, P < 0.01 compared between “pten null” and “WT EF only” groups; Additional file 10: Video S10), suggesting EF-triggered myosin posterior redistribution is PTEN dependent.

### Pten asymmetric redistribution is an early event preceded myosin II in electrotaxis

To further explore the spatial and temporal correlation between PTEN and myosin II during electrotaxis, we conducted a time-lapse analysis to exam the plasma membrane translocation of PTEN-GFP vs myosin II-GFP in WT cells under 10 V/cm EF. Posterior vs anterior GFP intensity ratio analysis was performed at all time points to elucidate the asymmetric translocation of the GFP signals toward the posterior of the electrotaxing cells. The higher the intensity ratio is, the more asymmetric posterior redistribution of GFP signals the cells generate. Both PTEN-GFP and myosin II-GFP showed a gradually increased intensity ratio for 80 s until they reached the maximum level in electrotaxing WT cells (Fig. 4a, red and blue lines).

Interestingly, the asymmetric membrane relocalization of PTEN-GFP in EF was an early event than the asymmetric recruitment of myosin II-GFP. In comparison with the PTEN-GFP intensity ratio elevation, which started from 42 ± 9.4-s post EF treatment (Fig. 4a, red line), there was ~ 60 s delay of the myosin II-GFP rear redistribution when treated with the same EF (Fig. 4a, blue line). The time-lapse immunoblotting analysis further confirmed that compared with phospho-PTEN (pS380) expression within the plasma membrane fraction, which became detectable from 120 s (Fig. 2k), the myosin II was only expressed on plasma membrane from 180-s post EF stimulation (Fig. 4h, i). EF-triggered membrane translocation and posterior redistribution of myosin II-GFP have fully abolished in *Pten* null cells (Figs. 3l-q; 4a, h, i, orange lines). Putting together, these data suggest that PTEN asymmetric activation might be an upstream regulator of myosin II in the electrotaxis of *Dictyostelium* cells.

Further time-lapse analysis comparing electrotactic response and PTEN/myosin II membrane translocation of the cells revealed that PTEN/myosin asymmetric redistribution were earlier events than the electrotactic response of the cells. The electrotactic response of cells initiated at 124 ± 8.3 s and peaked at 211 ± 10.4 s (Fig. 4b, red line), which is ~ 80 s or ~ 20 s behind the PTEN-GFP or myosin II-GFP membrane redistribution, respectively (Fig. 4a, red and blue lines).

To further support the causal link of the delayed myosin II redistribution downstream of PTEN rather than due to a slower accumulation of myosin II activation in EF, we temporarily blocked the asymmetric redistribution of PTEN-GFP & myosin II-GFP with LatA while maintaining their saturated activation with continuous EF treatment, then washed out LatA to restart the asymmetric redistribution analysis. LatA washout restored the EF-controlled posterior membrane translocation of PTEN-GFP from 122 ± 8.7 s and peaked at 181 ± 9.4 s (Fig. 4g, red line). In comparison, LatA washout also reinstated the posterior redistribution of myosin II-GFP in EF, but with ~ 60-s delay after PTEN-GFP (Fig. 4g, blue line).

Taken together, the spatial–temporal dynamic observation above suggests the causal link that electric signals triggered PTEN asymmetric plasma membrane translocation and activation, and subsequent myosin II posterior redistribution to regulate the electrotactic response of *Dictyostelium*.

### PTEN dependent anterior plasma membrane translocation of PH-Crac in EF

**Fig. 5:**
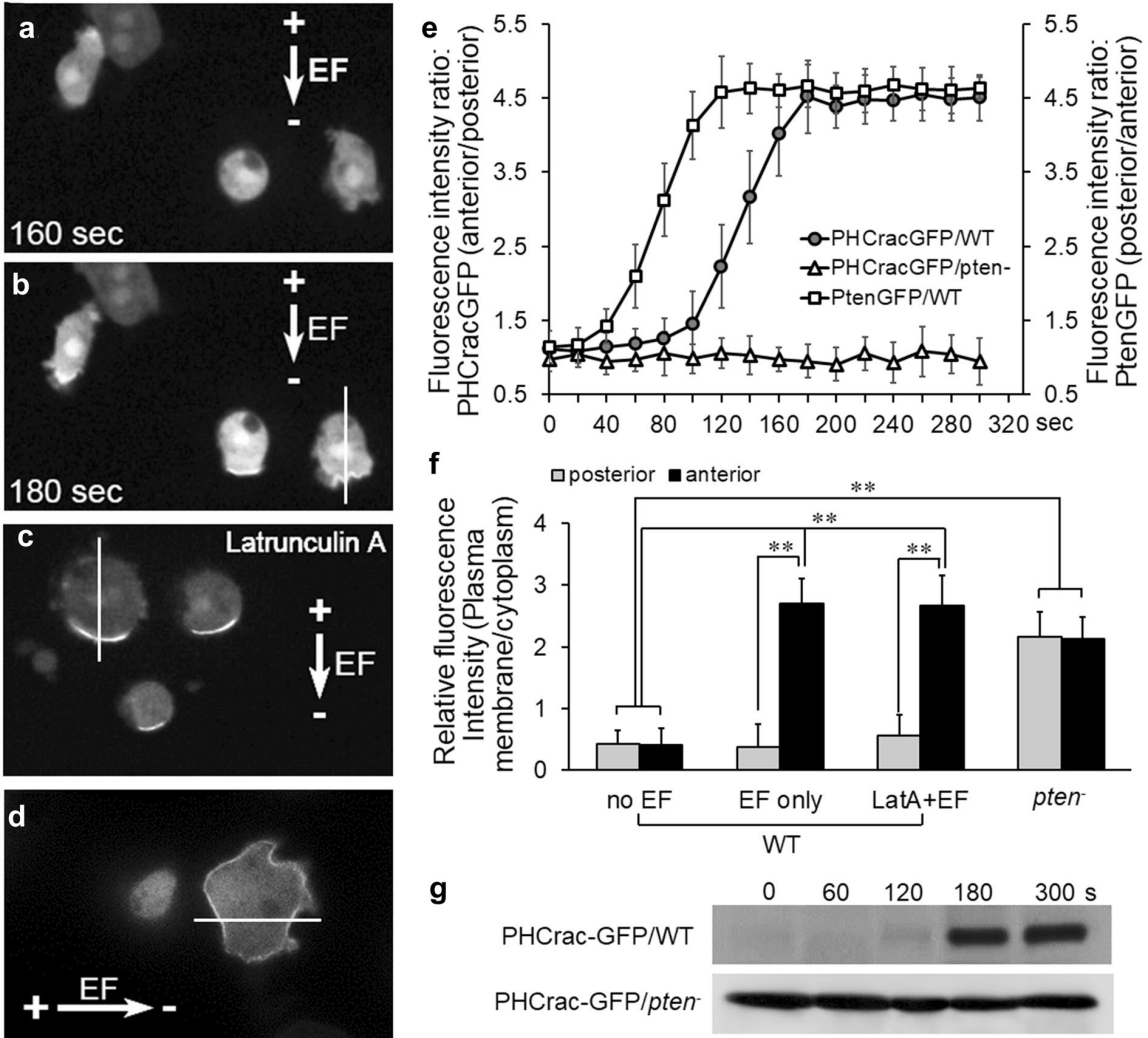
PTEN dependent anterior plasma membrane translocation of PH-Crac in EF. **a-b** PHCrac-GFP was redistributed asymmetrically to the leading edge of the electrotaxing WT cells. EF = 30 V/cm. **c** Latrunculin A treatment did not affect the EF-triggered asymmetric redistribution of PH-Crac in WT cells. **d** PH-Crac anterior relocalization was abolished in *pten*^*−*^ cells, while evenly distributed global membrane translocation remained. **e** Time-lapse analysis of fluorescence intensity ratio comparing membrane translocation of PTEN-GFP (posterior vs anterior) and PHCrac-GFP (anterior vs posterior) plasma membrane in EF. The asymmetric relocalization of PHCrac-GFP was fully abolished in *pten*^*−*^ cells. **f** Relative fluorescence intensity analysis further confirmed that the EF-triggered anterior redistribution of PHCrac-GFP was not affected by latrunculin A in WT cells, and significantly increased membrane translocation was recorded at both anterior and posterior of *pten*^*−*^ cells. **g** Membrane fraction cell lysates from the cells treated with the indicated duration of EFs were analyzed by immunoblotting with antibodies against PHCrac-GFP in WT or *pten*^*−*^ cells, respectively

Since PH-Crac binds to PIP3 and asymmetrically redistributed to the leading edge of migrating cells [39], we examined PHCrac-GFP expression as an indicator of PIP3 signaling in WT and *pten* null cells during electrotaxis. EF at 20 V/cm and above triggered electrotaxis of the cells with asymmetric recruitment of PHCrac-GFP to the anterior plasma membrane of electrotaxing cells (Fig. 5a, b; 5F, P < 0.01 compared between the anterior and posterior membrane of EF-treated WT cells; Additional file 11: Video S11). Interestingly, in contrast with the actin-dependent PTEN and myosin II posterior redistribution in EFs (Figs. 2d and 3f), PH-Crac anterior redistribution did not require actin polymerization when treated with LatA in EF (Fig. 5c, f, P < 0.01 compared between the anterior and posterior membrane of LatA-treated WT cells in EF; Additional file 1^11^ Video S11). The EF-triggered spatial–temporal dynamics of PHCrac-GFP was time-lapse recorded with time zero marked when EF was switched on (Additional file 12: Fig. S1). In wild type cells, the EF-induced electrotactic response started from 180 s onwards post EF exposure when asymmetric anterior relocalization of PHCrac-GFP was observed consistently (Additional file 12: Fig. S1a–c). LatA was applied from 460 s post EF exposure (Additional file 12: Fig. S1c). EF-induced PHCrac-GFP anterior membrane redistribution was sustained during the transition of actin depolymerization (Additional file 12: Fig. S1d-i). EF-triggered PH-Crac anterior redistribution is PTEN dependent since PH-Crac translocated evenly to the entire plasma membrane of *pten*^*−*^ cells in EF (Fig. 5d, f, p > 0.05 compared between the anterior and posterior membrane of EF-treated *pten*^*−*^ cells; Additional file 13: Video S12). When *pten*^*−*^ cells were exposed to LatA in EF, PHCrac-GFP continuously demonstrated even plasma membrane distribution, which was constantly observed prior (Additional file 14: Fig. S2a-c) and during (Additional file 14: Fig. S2d-i) actin depolymerization. Time-lapse analysis on GFP intensity ratio revealed that PH-Crac anterior redistribution peaked at ~ 180-s post EF treatment which was ~ 60 s later than PTEN-GFP posterior relocalization (Fig. 5e). This is in agreement with the membrane fraction immunoblotting analysis that PHCrac-GFP was only detectable from 180-s post EF treatment (Fig. 5g), compared with PTEN-GFP, which was shown from 120 s onwards in EF (Fig. 2k). These data further suggest that PTEN asymmetric redistribution might be an upstream event regulating PH-Crac activity in electrotaxis.

### PTEN mediated electrotaxis via maintenance of biased PHCrac-GFP expressing pseudopod protrusion toward the cathode

**Fig. 6:**
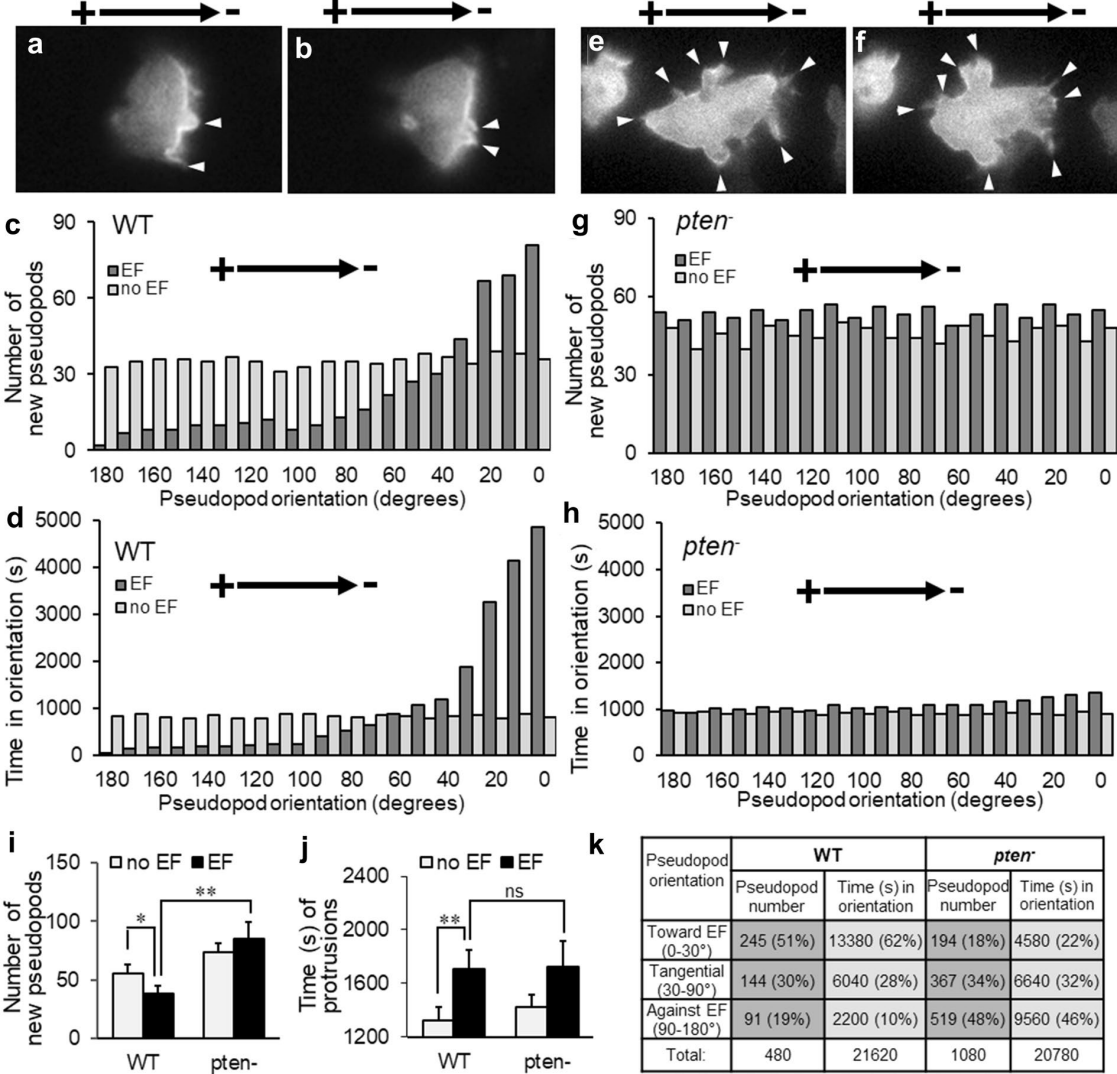
PTEN mediated electrotaxis via maintenance of biased pseudopod protrusion toward the cathodal forwarding direction. PHCrac-GFP containing pseudopod protrusions were quantified. **a-b** EF triggered a biased pseudopod redistribution at the anterior of electrotaxing WT cells. Two representative consecutive time points are shown. Time interval = 20 s. **c** and **g** Distribution of total pseudopods number of 12 EF-treated WT or *pten*^*−*^ cells, respectively. **d** and **h** Distribution of total time spent in each category from pseudopods of 12 EF-treated WT or *pten*^*−*^ cells, respectively. **e** and **f** EF-triggered pseudopod anterior redistribution was fully abolished in *pten*^*−*^cells. Two representative consecutive time points are shown. Time interval = 20 s (**i**) Comparing the average new pseudopods number generated between a single WT and *pten*^*−*^ cell, in the absence or presence of EF for 320 s. **j** Comparing total pseudopods time generated between a single WT and *pten*^*−*^ cell, in the absence or presence of EF for 320 s. **k** Comparison studies of pseudopod number and time in orientation of 12 EF-treated WT or *pten*^*−*^ cells, respectively. Recording time = 320 s. All data are confirmed from minimal 3 independent experiments

EF triggered a biased distribution of PHCrac-GFP positive pseudopod protrusion in the anterior region of the electrotaxing cells (Fig. 6). Compared with the no EF control group, which showed randomly distributed pseudopod protrusions (Fig. 6c), there was a clear shift of the newly formed PHCrac-GFP pseudopods at the anterior region of the migrating WT cells toward cathode (Fig. 6a-c). We recorded 51% of the total pseudopods orientating toward EF vector (0–30 degree) compared with 18% in the control group (0–30 degree; Fig. 6k). Interestingly, compared with the pseudopod production rate of no-EF WT control cells at 23 ± 5.1 s per pseudopod (s/ps), EF-triggered a much longer lifetime of the PHCrac-GFP positive pseudopods at the forwarding direction in EF (0–30 degrees) than the rest of the orientations (Fig. 6d), with a much higher average production rate at 55 ± 6.2 s/ps within 0–30 degrees toward EF than the protrusions against EF (90–180 degrees) at 24 ± 5.5 s/ps (Fig. 6k). This is in sharp contrast with the chemotaxing cells, which showed a uniform pseudopod production rate at ~ 23 s/ps irresponsive to chemoattractant gradient [54]. More strikingly, on the contrary to the speculation that EF might have promoted more pseudopods to expedite the sharp membrane translocation of myosin/F-actin and increased motility in electrotaxis, our data demonstrated that EF significantly suppressed the total number of PHCrac-GFP pseudopod protrusions (Fig. 6i, P < 0.05), while significantly increased the total protrusion lifetime compared to no EF control (Fig. 6j, P < 0.01). These data above suggested that EF persistently maintains more persistent PHCrac-GFP expressing pseudopods in the forwarding direction rather than generates an increased number of protrusions to facilitate the electrotactic response.

In contrast with WT cells (Fig. 6a–d), *pten*^*−*^ cells completely lost biased redistribution of PHCrac-GFP positive pseudopods in EF, as shown by the evenly distributed protrusions across all orientations (Fig. 6e–g). Compared with WT cells which had 51% PHCrac-GFP pseudopods concentrated in the forwarding direction in EF (0–30 degrees), there was a significantly reduced proportion of protrusions with forwarding orientation (18%) in *pten*^*−*^ cells when treated with the same EF (Fig. 6k). On the other hand, WT cells showed significantly reduced PHCrac-GFP positive pseudopods at the posterior regions of the electrotaxing cells (19%; Fig. 6k), while *pten* knockout produced evenly distributed PHCrac-GFP pseudopods and significantly more pseudopods at the posterior regions of the cells (48%; Fig. 6k), with the total protrusion number more than doubled compared with WT cells (Fig. 6I, P < 0.01; Fig. 6k).

Interestingly, EF-promoted protrusions in *pten*^*−*^ cells are short-lived. Compared with the average lifetime of WT pseudopods of all orientation at 45 ± 5.8 s, *pten*^*−*^ cells showed a significantly reduced average lifetime at 20 ± 5.9 s (Fig. 6k). This was further confirmed when comparing the average lifetime of pseudopods in the forwarding direction (0–30 degrees) of EF: *pten*^*−*^ cells showed a reduced average lifetime of pseudopods at 24 ± 4.8 s/ps compared with WT cells at 55 ± 6.5 s/ps in the forwarding direction (Fig. 6k). As a combined effect of the observations above, EF triggered a significantly increased overall lifetime of PHCrac-GFP pseudopods facing 0–30 degrees of electrotaxing direction (62% or 13,380 s), which was notably diminished by *pten* knockout (22% or 4580 s). On the other hand, EF significantly suppressed the overall PHCrac-GFP pseudopod lifetime in the posterior region of the WT cells (10% or 2200 s) compared with that of *pten*^*−*^ cells (46% or 9560 s). These data above is in harmony with the dynamic changes of pseudopod protrusion length: EF-triggered significantly reduced pseudopod length at the posterior region, and increased protrusion length at the anterior area of the electrotaxing cells (Fig. 4a: black = anterior; green = posterior).

### PTEN promoted anterior redistribution of F-actin colocalized with PH-Crac signaling

**Fig. 7:**
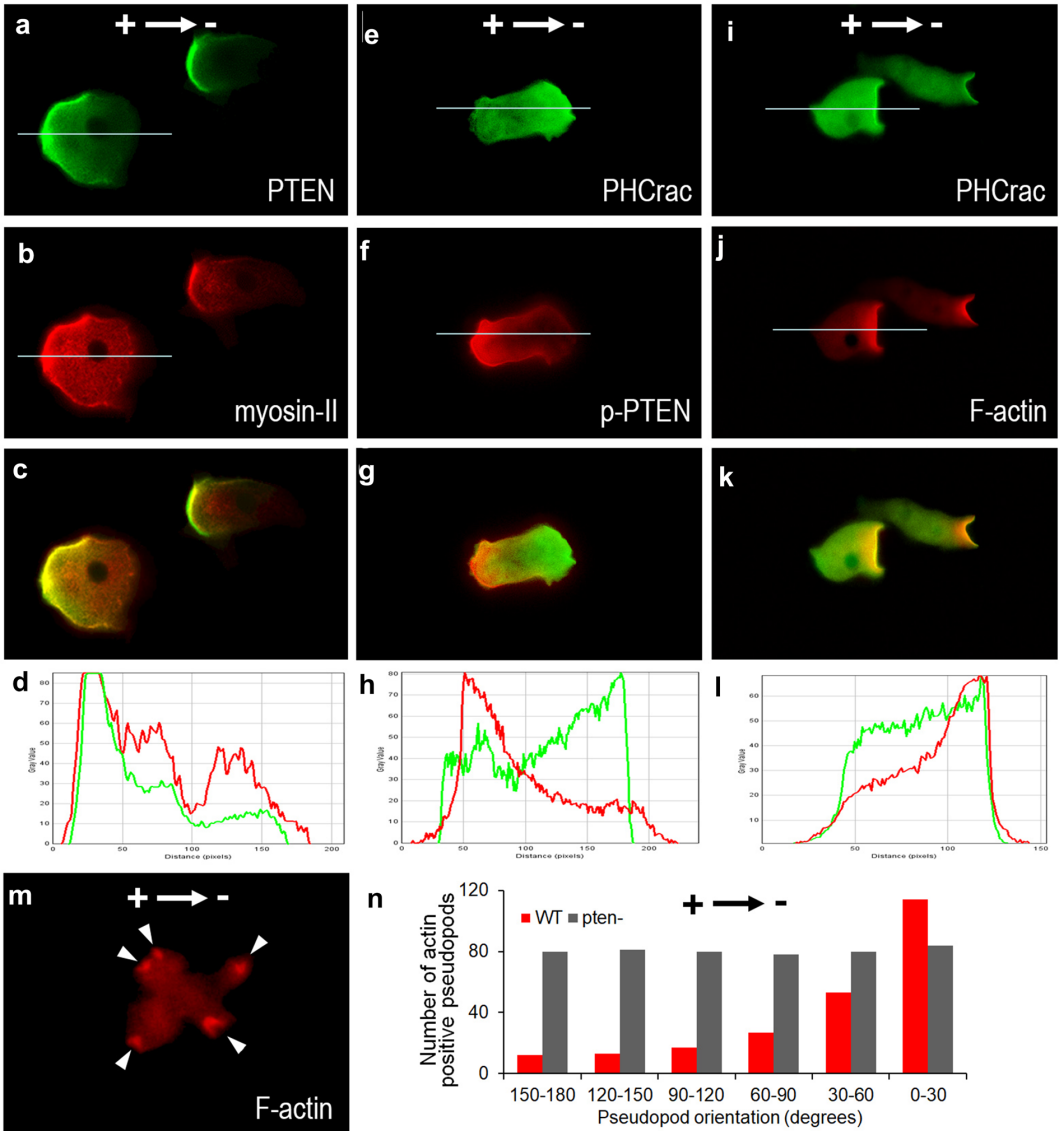
Immunocytochemistry staining for PTEN, myosin II, PH-Crac and F-acin in EF. **a–d** PTEN-GFP and myosin II colocalized at the posterior plasma membrane of the electrotaxing cells. **e–h** Phospho-PTEN and PHCrac-GFP asymmetrically redistributed to the posterior and anterior membrane of the electrotaxing cells, respectively. **i–l** PHCrac-GFP and F-actin colocalized at the anterior plasma membrane of the electrotaxing cells. **m** F-actin leading-edge recruitment was abolished in pten- cells. **n** Distribution of F-actin positive pseudopods number of EF-treated WT or *pten*^*−*^ cells, respectively

EF triggered posterior colocalization of myosin and phospho-PTEN (Fig. 7a–d). Phospho-PTEN and PH-Crac localized in the opposite directions toward anode and cathode, respectively (Fig. 7e–h). At the same time, EF also triggered anterior redistribution of F-actin, which colocalized with PH-Crac at the leading edge of electrotaxing cells (Fig. 7i–l). *Pten* knockout fully abolished the F-actin redistribution response to EF treatment (Fig. 7m), with an evenly distributed number of pseudopods across all directions (Fig. 7n, grey columns) compared with the anterior relocalization of WT pseudopods (Fig. 7n, red columns).

## Discussion

PTEN has a central role in association with the PI3K network to regulate cell migration in both lower and higher eukaryotes [23]. We demonstrated in this study that the asymmetric translocation of activated PTEN at the posterior plasma membrane of cells is essential in the regulation of electrotaxis, which in turn triggers myosin II posterior membrane recruitment and PIP3/F-actin anterior relocalization to facilitate such response.

### PTEN signaling is essential in the regulation of electrotaxis of *Dictyostelium*

In contrast with chemotaxis, the electrotactic defect of *pten*^*−*^ cells could be fully rescued when treated with higher EF at 30 V/cm. It is worth noting that such restoration of electrotactic response by higher EFs requires a significantly longer time than WT cells, as revealed by the time-lapse analysis of migration directedness in EFs (Fig. 4b). This suggests the possibility that an alternative signaling pathway might exist (at least at higher EFs) to coordinate electrotaxis with PTEN together; thereby, accumulative negative feedback might kick in in the absence of PTEN signals to compensate the lost electrotactic response in *pten*^*−*^ cells.

Interestingly, our current observation is in contrast with our previous study on mammalian cells that loss of PTEN markedly enhanced EF-induced monolayer wound healing with increased keratinocyte migration speed and directedness [9]. Several studies have indicated that the mechanism of mammalian PTEN in the regulation of cell migration is rather different from that of *Dictyostelium*. Several lineages of genetic modified *pten* null mammalian cells migrate faster than wild-type cells [19, 20]. As *pten*^*−*^ cells migrate almost twice as quickly as wild type cells, thus the wound of *pten* null fibroblasts heals twice as fast as wild type cells [19].

Indeed, the detailed roles for PTEN to regulate cell migration appears to depend on cell types. Some chemoattractant signal amplification mechanisms are not conserved between *Dictyostelium* and mammalian cells. PTEN exhibits random recruitment to the plasma membrane in resting Dictyostelium cells, whereas in mammalian cells, PTEN is mostly observed in the cytosol. In mammalian cells, PTEN negatively regulates cell motility by downregulation of Rac1 and Cdc42 activation in vitro. And this downregulation is dependent on the lipid phosphatase activity of PTEN [19]. However, some other studies indicated that the activity of protein phosphatase of PTEN could also inhibit the migration of tumor cells [55]. A previous report reveals that the inhibition of PTEN knockout human glioma cell migration is mediated by PTEN C2 domain phosphorylation, which is lipid phosphatase activity independent [56]. PTEN controls actin cytoskeleton remodeling in mammalian cells chemotaxis either through its lipid or protein phosphatase activities [56]. However, another study indicates that PI(4,5)P_2_ binding domain at the N terminal of PTEN is critical for *Dictyostelium* chemotaxis [57].

These different responses between mammalian and *Dictyostelium* cells toward the genetic disruption of PTEN might help to explain the different electrotactic response of different cell types. It is also worth noting that EF-induced PTEN posterior membrane redistribution is actin polymerization dependent since LatA treatment abolished PTEN asymmetric relocalization as demonstrated in this study. This is in contrast with chemotaxis whereby cAMP gradient triggered PTEN posterior membrane redistribution in an actin polymerization independent manner [58].

### The possible crosstalk between PTEN and myosin-II in the control of electrotaxis

It is well accepted that the posterior translocation of myosin II is required for the retraction of the rear part of the directionally migrating cells, therefore biasing the direction of migration by repressing extensions of posterior and lateral pseudopodia [59–62]. Further studies indicate that PTEN is an upstream signaling component to relocalize myosin II, which is necessary for the suppression of lateral pseudopod formation [42, 43].

In the current study, we demonstrated an identical redistribution pattern of myosin II and PTEN, which colocalize at the posterior plasma membrane of electrotaxing cells, which is in agreement with chemotaxing cells [37, 47, 60]. In our electrotaxis assay, we clearly observed that the membrane relocalization of PTEN-GFP took place before that of myosin II in EF, and that both events preceded the electrotactic response of the cells, suggesting PTEN functions as a potential upstream activator of myosin II to regulate electrotaxis. The study also concludes that PTEN mediates the relocalization of F-actin/myosin II in the cell cortex, which is essential for suppression of posterior and lateral pseudopod formation through the generation of cortical tension [43]. Firtel and colleague reported that hyper-assembly of myosin II and F-actin was mediated by constitutive activation of PAKa [63]. In chemotaxing *Dictyostelium* cells, PAKa is localized at the posterior of the cells, which leads to myosin II assembly at the same site [59]. Although in mammalian neutrophils, PAK1 is localized at the leading edge of migrating cells upon stimulation of chemoattractants [64]. One of the possible explanations suggested by the Firtel group is that phosphorylated PAKa initially takes place at the leading edge of *Dictyostelium* cells, which allows interaction with other proteins and then travel to the rear of the cells to promote myosin II localized assembly [59]. On the other hand, in mammalian myeloid cells, Gβγ binds to P21-activated kinase 1 (PAK1), and activates cdc42 via PAK associated guanine nucleotide exchange factor (PIXα), which in turn excludes PTEN from the leading edge, promotes F-actin localized activation at the leading edge of cells, and ultimately triggers directional sensing and migration [36]. These observations might also help to understand the PTEN–myosin II correlation, and the different roles of PTEN in the regulation of electrotaxis of mammalian and *Dictyostelium* cells: In mammalian cells, Gβγ-PAK1/PIXα/Cdc42 pathway excludes PTEN from the leading edge of cells and promotes localized F-actin formation at the leading edge of the cells, which consequently triggers the electrotactic response, whereby PTEN plays as a negative regulator in this event; In contrast, in *Dictyostelium* cells, PTEN redistribution to the posterior of the cells promotes myosin II localized assembly, thereby positively regulate electrotactic response (Additional file 15: Fig. S3).

### PTEN requirement in anterior signaling of PIP3/F-actin during electrotaxis

In chemotaxis, PTEN disassociates from the leading edge and is present only at the back and lateral side of the cells, hence restricting PtdIns(3,4,5)P_3_ accumulation only at the front of cells to facilitate chemotaxis. Loss of PTEN results in elevated, unregulated PtdIns(3,4,5)P_3_ production along the entire plasma membrane, hence the directional migration defect [16, 21, 57].

Actin polymerization showed a large but short crescendo followed by a rapid decrease in chemoattractant; *pten*^*−*^ cells, however, showed a significantly larger and longer actin polymerization sixfold higher than WT cells [16]. We demonstrated in this study that EF triggered extensively long duration of PHCrac-GFP membrane translocation to the leading edge of electrotaxing cells, which was a delayed response after PTEN asymmetric redistribution. However, this phenomenon requires a much higher EF voltage to facilitate compared to that of PTEN/myosin, which is in agreement with our previous finding that lower EF did not trigger anterior recruitment of PHCrac-GFP [7]. EF-induced PHCrac-GFP anterior membrane relocalization is actin polymerization independent, as shown with persistent leading edge recruitment when exposed to LatA. This is in consistent with cAMP gradient-induced PHCrac-GFP asymmetric redistribution when actin is depolymerized with latrunculin treatment [58].

PH-Crac membrane relocalization occurred at exactly the same time as myosin II asymmetric recruitment, immediately preceded the initiation of the electrotactic response. One explanation could be that EF triggered PTEN posterior redistribution and suppressed the anterior relocalization of PTEN, thereby maintained a lower PIP3 through phosphatase function at the rear region of the cells, and a subsequent higher PIP3 accumulation at the leading edge of the electrotaxing cells. Therefore PH-Crac was released from the posterior membrane and bonded to PIP3 dominantly at the leading edge of the electrotaxing cells. This could also explain the persistent global activation of PIP3 across the entire plasma membrane, attracting prolonged PH-Crac binding evenly across the entire membrane of *pten*^*−*^ cells in EF, and the subsequent F-actin colocalization at the leading edge of the electrotaxing cells (Additional file 15: Fig. S3).

### PTEN is required for the actin redistribution and biased pseudopod production to facilitate electrotactic response in *Dictyostelium*

A previous study by Insall and colleague reported that the pseudopod generation of chemotaxing *Dictyostelium* was independent of chemotactic signaling, and the new pseudopod formation was made at the same rate regardless of the chemoattractant gradient [54]. The directional sensing in chemotaxis was facilitated through selective maintenance of the most accurate pseudopod toward gradients [54]. In contrast with the chemotaxis, we showed in this study that EF triggered a dramatic increase of the pseudopod production rate in the forwarding direction, which was more than doubled compared with the protrusions facing against EF direction. The fact that EF also triggered a significant reduction of the WT cells pseudopod number, suggesting that EF facilitates electrotaxis by suppressing inefficient pseudopods in the “wrong” direction against EF to avoid the internal competition of random protrusions against each other, and promoting the survival of the pseudopods in the “correct” migration direction toward cathode of the EF. This event is PTEN dependent since comparing with the reduced number and longer production rate of the pseudopod from WT cells, *pten* knockout surprisingly showed more than doubled pseudopods in EF with much reduced lifetime. In combination with the observation that PTEN-GFP promoted F-actin leading-edge colocalization with PH-Crac in EF, we conclude that PTEN facilitates EF-triggered asymmetric redistribution of posterior myosin and anterior F-actin, thereby promotes significantly reduced posterior pseudopod formation and elevated anterior protrusion in the EF direction. However, the mechanism of possible alternative regulators compensated the electrotactic response under higher EFs in *pten*^*−*^ cells remains elusive. A very interesting possible alternative mechanism is suggested by the results to restore electrotactic response at higher EFs in *pten*^*−*^ cells. Precise control of voltages in electrotaxis experiments will offer practical approach to elucidate the mechanism in the future.

## Supplementary Information

Additional file 1: **Video S1.** EFs triggered an obvious electrotactic response of Dictyostelium cells toward the cathode.

Additional file 2: **Video S2.** Pten- cells showed significantly reduced electrotactic response.

Additional file 3: **Video S3.** PTEN re-expression in pten- cells completely reinstated the reduced electrotactic response of pten- cells.

Additional file 4: **Video S4.** EF stimulation triggered significant posterior plasma membrane translocation of PTEN-GFP

Additional file 5: **Video S5.** Latrunculin A (LatA) treatment completely abolished the EF-triggered PTEN posterior plasma membrane redistribution and electrotaxis of the cells.

Additional file 6: **Video S6.** Washout LatA fully restored the posterior plasma membrane translocation of PTENGFP in EF

Additional file 7: **Video S7.** EF triggered posterior redistribution of myosin II to plasma membrane recruitment electrotaxing Dictyostelium.

Additional file 8: **Video S8.** LatA-treatment abolished EF-triggered asymmetric redistribution of myosin II-GFP.

Additional file 9: **Video S9.** Washout of latA fully restored the asymmetrical redistribution of myosin II-GFP to the posterior plasma membrane of the electrotaxing cells.

Additional file 10: **Video S10.** Pten null cells lost posterior membrane translocation of myosin II-GFP in EF.

Additional file 11: **Video S11.** Higher EF at 20 V/cm and above triggered electrotaxis of the cells with asymmetric recruitment of PHCrac-GFP to the anterior plasma membrane of electrotaxing cells, which did not require actin polymerization when treated with LatA in EF.

**Additional file 12: Figure S1.** EF-induced PHCrac-GFP anterior plasma membrane translocation is independent of actin polymerization. PHCrac-GFP was redistributed asymmetrically to the leading edge of the electrotaxing WT cells (**a-c**). LatA was applied at 460-sec post EF treatment, and actin polymerization was fully abolished at 860-sec post EF treatment. PHCrac-GFP anterior redistribution was consistently observed throughout LatA exposure (**d-i**).

Additional file 13: **Video S12.** PH-Crac translocated evenly to the entire plasma membrane of pten- cells in EF.

**Additional file 14: Figure S2.** EF-induced PHCrac-GFP anterior plasma membrane translocation was abolished in pten null cells.** a-c** In the absence of LatA, PHCrac-GFP was distributed evenly to the plasma membrane of pten null cells in EF. LatA was applied at 240-sec post EF treatment, and actin polymerization was abolished at 660-sec post EF treatment.** d-i** EF-treated PTEN null cells were recorded in LatA. PHCrac-GFP cell membrane random distribution was consistently observed throughout the LatA treatment.

**Additional file 15: Figure S3.** The schematic diagram illustrates the PTEN-driven coordination of the phospho-PTEN/myosin (posterior) vs PIP3/F-actin (anterior) signaling during electrotaxis. EF stimulation triggered PTEN phosphorylation, which in turn dephosphorylates PIP3 to PIP2 and promotes the asymmetric redistribution of Myosin-II and p-PTEN to the posterior plasma membrane of the electrotaxing cells. At the same time, EF also promotes PIP3 phosphorylation anterior redistribution together with F-actin via PI3K activation.

## Data Availability

All data and materials are available via BS or MZ.
