# Correction to: Electric signals counterbalanced posterior vs anterior PTEN signaling in directed migration of Dictyostelium

**DOI:** 10.1186/s13578-021-00653-x

**Published:** 2021-07-21

**Authors:** Bing Song, Yu Gu, Wenkai Jiang, Ying Li, Wayne Nishio Ayre, Zhipeng Liu, Tao Yin, Christopher Janetopoulos, Miho Iijima, Peter Devreotes, Min Zhao

**Affiliations:** 1 School of Dentistry, College of Biomedical and Life Sciences, Cardiff University, CF14 4XY Cardiff, UK; 2 State Key Laboratory of Military Stomatology, Department of Operative Dentistry & Endodontics, School of Stomatology, Fourth Military Medical University, Xi’an, China; 3 Chinese Academy of Medical Sciences & Peking Union Medical College Institute of Biomedical Engineering, Tianjin, China; 4 BioImaging Core Facility, University of Sciences, Philadelphia, PA 19104 USA; 5 School of Medicine, Johns Hopkins University, Baltimore, MD 21205 USA; 6 Department of Ophthalmology & Vision Science, School of Medicine, UC Davis, CA 95618 Davis, USA; 7 Department of Dermatology, School of Medicine, University of California, Davis, CA 95618 USA

## Correction to: Cell Biosci (2021) 11:111 https://doi.org/10.1186/s13578-021-00580-x

In this article, the affiliation ‘Department of Ophthalmology & Vision Science, UC Davis, School of Medicine, Davis CA 95618, USA’ for Author Min Zhao was missing.

The affiliation has been updated above and the original article [1] has been corrected.

## References

[1] Song B, Gu Y, Jiang W, Li Y, Ayre WN, Liu Z, Yin T, Janetopolous C, Iijima M, Devreotes P, Zhao M (2021). Electric signals counterbalanced posterior vs anterior PTEN signaling in directed migration of Dictyostelium. Cell Biosci.

