# Supplementary figures and images for: Electric signals counterbalanced posterior vs anterior PTEN signaling in directed migration of Dictyostelium
Source: Cell Biosci. 2021 Jun 14;11:111. doi: 10.1186/s13578-021-00580-x (PMC8201722; doi:10.1186/s13578-021-00580-x)

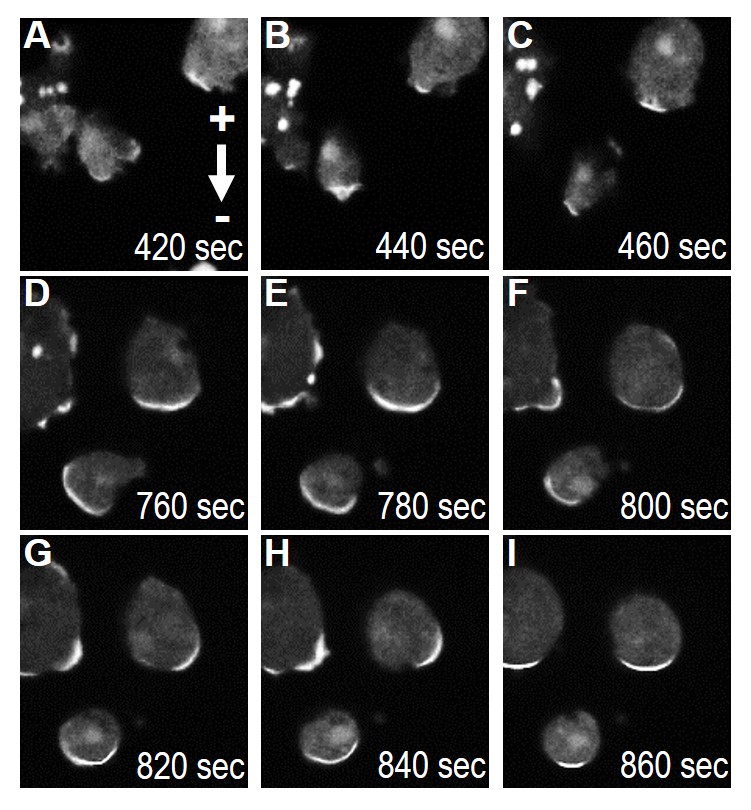

Supplement: Supplementary file 12 — Additional file 12: Figure S1. EF-induced PHCrac-GFP anterior plasma membrane translocation is independent of actin polymerization. PHCrac-GFP was redistributed asymmetrically to the leading edge of the electrotaxing WT cells (a-c). LatA was applied at 460-sec post EF treatment, and actin polymerization was fully abolished at 860-sec post EF treatment. PHCrac-GFP anterior redistribution was consistently observed throughout LatA exposure (d-i). [file 13578_2021_580_MOESM12_ESM.jpg]

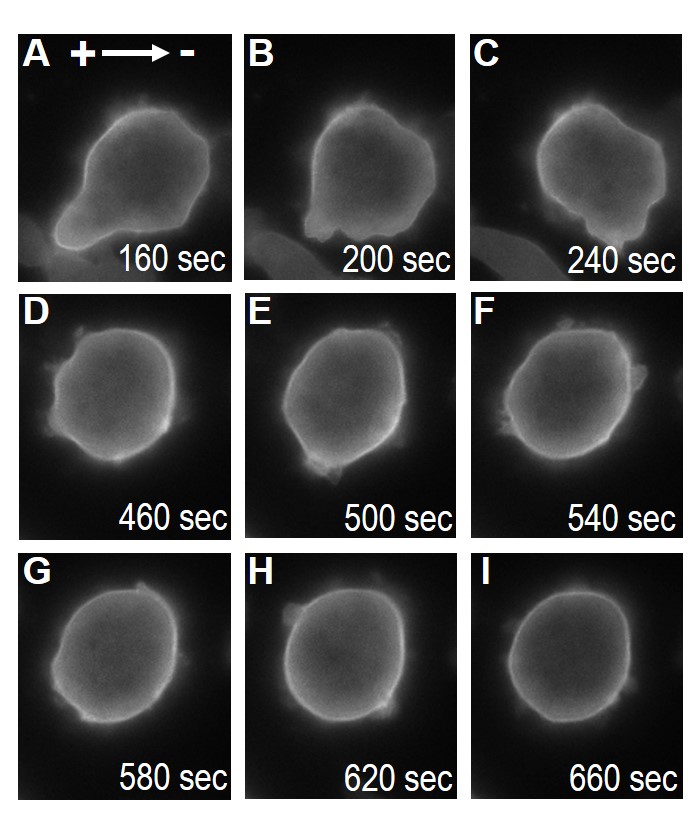

Supplement: Supplementary file 14 — Additional file 14: Figure S2. EF-induced PHCrac-GFP anterior plasma membrane translocation was abolished in pten null cells. a-c In the absence of LatA, PHCrac-GFP was distributed evenly to the plasma membrane of pten null cells in EF. LatA was applied at 240-sec post EF treatment, and actin polymerization was abolished at 660-sec post EF treatment. d-i EF-treated PTEN null cells were recorded in LatA. PHCrac-GFP cell membrane random distribution was consistently observed throughout the LatA treatment. [file 13578_2021_580_MOESM14_ESM.jpg]

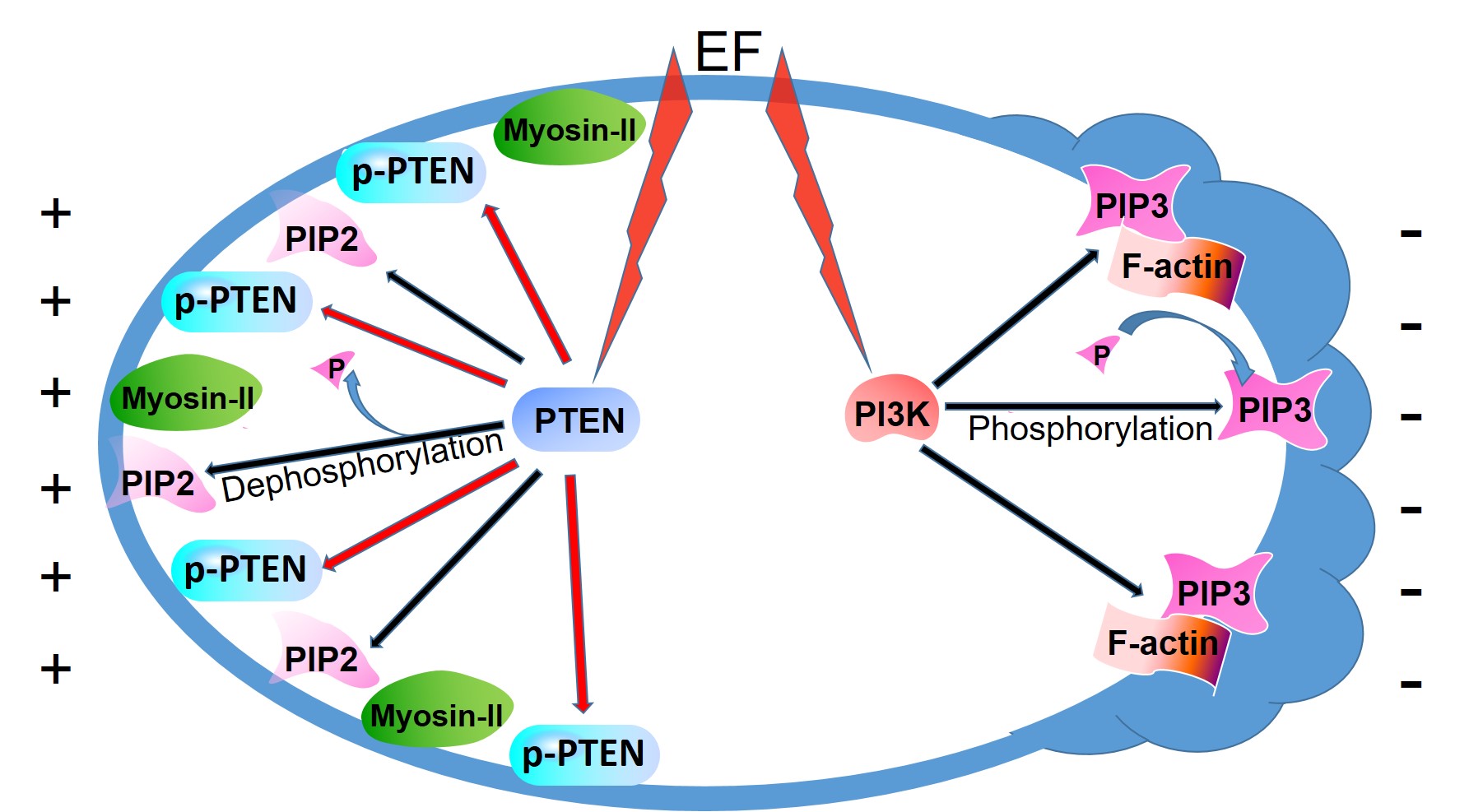

Supplement: Supplementary file 15 — Additional file 15: Figure S3. The schematic diagram illustrates the PTEN-driven coordination of the phospho-PTEN/myosin (posterior) vs PIP3/F-actin (anterior) signaling during electrotaxis. EF stimulation triggered PTEN phosphorylation, which in turn dephosphorylates PIP3 to PIP2 and promotes the asymmetric redistribution of Myosin-II and p-PTEN to the posterior plasma membrane of the electrotaxing cells. At the same time, EF also promotes PIP3 phosphorylation anterior redistribution together with F-actin via PI3K activation. [file 13578_2021_580_MOESM15_ESM.jpg]
